# DNA methylation analysis of porcine mammary epithelial cells reveals differentially methylated loci associated with immune response against *Escherichia coli* challenge

**DOI:** 10.1186/s12864-019-5976-7

**Published:** 2019-07-31

**Authors:** Basavaraj Sajjanar, Nares Trakooljul, Klaus Wimmers, Siriluck Ponsuksili

**Affiliations:** 1 Leibniz Institute for Farm Animal Biology (FBN), Institute for Genome Biology, Functional Genome Analysis Research Unit, Wilhelm-Stahl-Allee 2, D-18196 Dummerstorf, Germany; 2 Leibniz Institute for Farm Animal Biology (FBN), Institute for Genome Biology, Genomics Research Unit, Wilhelm-Stahl-Allee 2, D-18196 Dummerstorf, Germany

**Keywords:** DNA methylations, Coliform mastitis, Immune response, CpG islands

## Abstract

**Background:**

Epigenetic changes such as cytosine (CpG) DNA methylations regulate gene expression patterns in response to environmental cues including infections. Microbial infections induce DNA methylations that play a potential role in modulating host-immune response. In the present study, we sought to determine DNA methylation changes induced by the mastitis causing *Escherichia coli* (*E. coli*) in porcine mammary epithelial cells (PMEC)*.* Two time points (3 h and 24 h) were selected based on specific transcriptomic changes during the early and late immune responses, respectively.

**Results:**

DNA methylation analysis revealed 561 and 898 significant (*P <* 0.01) differentially methylated CpG sites at 3 h and 24 h after *E. coli* challenge in PMEC respectively. These CpG sites mapped to genes that have functional roles in innate and adaptive immune responses. Significantly, hypomethylated CpG sites were found in the promoter regions of immune response genes such as *SDF4, SRXN1, CSF1* and *CXCL14*. The quantitative transcript estimation indicated higher expression associated with the DNA CpG methylation observed in these immune response genes. Further, *E. coli* challenge significantly reduced the expression levels of *DNMT3a*, a subtype of de novo DNA methylation enzyme, in PMEC indicating the probable reason for the hypomethylation observed in the immune response genes.

**Conclusions:**

Our study revealed *E. coli* infection induced DNA methylation loci in the porcine genome. The differentially methylated CpGs were identified in the regulatory regions of genes that play important role in immune response. These results will help to understand epigenetic mechanisms for immune regulation during coliform mastitis in pigs.

**Electronic supplementary material:**

The online version of this article (10.1186/s12864-019-5976-7) contains supplementary material, which is available to authorized users.

## Background

Epigenomic changes are dynamically regulated by environmental cues. Among the various epigenetic modifications, cytosine (CpG) methylation of genomic DNA is an important reversible gene regulatory mechanism. DNA methylation plays a crucial role in transcriptional regulation by affecting the recruitment of regulatory factors onto promoters and enhancers [1]. Microbial infections, where induced DNA methylation may modulate host immune responses [2, 3]. It has been shown that human papillomavirus infection cause aberrant host DNA methylation through direct interaction of viral protein E7 with DNA methyltransferase 1 (*DNMT1*) [4]. *Escherichia coli* infection (UPEC) of human uroepithelial cells was reported to regulate the expression of *DNMT1,* and CpG hypermethylation down-regulated the cell-cycle inhibitor *CDKN2A* likely inhibiting apoptosis and increasing proliferation of uroepithelial cells [5]. Infection by the protozoan, *Leishmania donovani* also alters DNA methylation profiles in human macrophages suppressing the host immune response and enabling intracellular survival of the protozoan [6]. Similar observations were made for *Mycobacterium tuberculosis* (TB) infection that rapidly methylates host DNA at distal enhancer elements and associated chromatin remodelling [7]. Schistosoma parasite infection induces hypermethylation in transcription factors that inhibit IFN-γ signalling in CD4+ T cells of children, with a significant effect on the downstream TB-specific immune phenotypes [8]. These epigenetic effects of bacterial and parasitic infections and subsequent regulations of immune response highlight the role of DNA methylations in host-pathogen interactions.

Coliform mastitis (CM) causes postpartum dysagalactia syndrome (PDS), an important disease in pigs. The affected animals show high fever, loss of appetite, pain and inflammation of teats. PDS is a disease of economic significance as it severely affects the health and milk production of sows leading to poor survival of piglets [9]. Gram-negative bacteria such as *Escherichia coli* (*E. coli*) are the most prominent causative pathogens isolated from PDS- affected sows. Lipopolysaccharide (LPS), an outer membrane endotoxin component of *E. coli,* is the major pathogenic factor that can induce inflammatory responses in sows with PDS [10]. In our previous studies, an *E. coli* bacterial challenge of porcine mammary epithelial cells (PMECs) was used as a model for porcine mastitis at two time points (3 h and 24 h) that represented early and late transcriptional responses [11]. There was a distinct transcription pattern at 3 h and 24 h post challenge of *E. coli* in porcine mammary epithelial cells and found upregulated set of genes, including of cytokines, chemokines, and cell adhesion factors, which together coordinate the immune response of host cells [11]. These time points broadly represented early and late transcriptional responses. These changes are mediated through epigenetic mechanism including microRNA [12]. We hypothesized that the *E. coli* induced transcriptomic changes in PMEC also follow other epigenomic modifications. Therefore, our present study focuses on host-cell DNA methylation changes induced by the mastitis causing *E. coli.*

## Results

### Genome-wide DNA methylation in PMEC

Genome-wide DNA methylation profiling of PMEC (unchallenged control, 3 h post-challenge (hpc) *E. coli* and 24hpc *E. coli*) generated 25–40 million Illumina sequencing reads for each of the nine reduced-representation bisulfite sequencing (RRBS) libraries. Mapping to the pig genome (*Sscrofa* 11.1) using Bismark (Bowtie 2) revealed that overall 50% of the generated reads uniquely mapped to the genome. Twenty percent of the CpGs analysed across all samples mapped to the 5′ regulatory region (promoter) of genes with functional annotations. Further, approximately 45% of mapped CpGs were enriched in known CpG islands of the pig genome. The average CpG methylation levels was approximately 45% and the non-CpG level was less than 10% in both the control and treated groups (Table 1, Additional file 1: Figure S1). The analysis of methylation levels of the CpG sites in different regions of the genome indicated that CpGs located in the upstream promoter regions of genes had lower methylation levels (< 10%); whereas, gene bodies and intragenic spaces had higher methylation levels (> 30%). In total, 49,921 CpG positions which for common across all the control and treated samples were identified for further study after quality checks and normalization with at least 10X coverage. These methylation levels were similar and no significant differences were observed across different samples. Further, the methylation levels of the CpGs presented a similar bimodal distribution in all the samples of control and treatment, which is consistent with the results of earlier studies on distribution of CpG methylation (Additional file 2: Figure S2).

**Table 1:** Details of mapping of RRBS libraries to the porcine genome (Sscrofa11.1) using Bismark (Bowtie 2)

Sample ID	Clean reads	Unique alignments	Mapping efficiency	CpG methylation	Non-CpG methylation
Control_1	24181312	12123248	50.10%	46.10%	7.30%
Control_2	32044506	15725669	49.10%	44.60%	9.10%
Control_3	29982045	15230863	50.80%	45.90%	7.80%
*E. coli*_3hpc1	30859633	15493727	50.20%	46.60%	7.50%
*E. coli_*3hpc2	34963148	17567402	50.20%	45.40%	8.00%
*E. coli_*3hpc3	23771692	11990617	50.40%	46.10%	8.30%
*E. coli_*24hpc1	42369408	21430556	50.60%	46.70%	8.20%
*E. coli_*24hpc2	28519930	13814779	48.40%	44.90%	7.40%
*E. coli*_24hpc3	29039945	15042857	51.80%	45.50%	7.70%

### Differentially methylated CpGs in *E. coli* challenged PMEC

After quality checks and normalization with at least 10X coverage by using pairwise analysis, 82,833, 132,624 and 116,632 CpG positions common between control and *E. coli* 3 hpc group, between control and *E. coli* 24 hpc group and between *E. coli* 3 hpc group and 24 hpc group, respectively, were further used for differential methylation analysis. There were a total of 561 differentially methylated CpGs when comparing the *E. coli* 3 hpc group and the unchallenged control group (*P* < 0.01, Additional file 6). A total of 898 differentially methylated CpGs when comparing the *E. coli* 24 hpc group and unchallenged control group (*P* < 0.01, Additional file 7). The analysis between 3 hpc and 24 hpc groups indicated 855 differentially methylated CpGs loci (*P* < 0.01, Additional file 8) and their similar distribution in different genomic features.

**Fig. 1:**
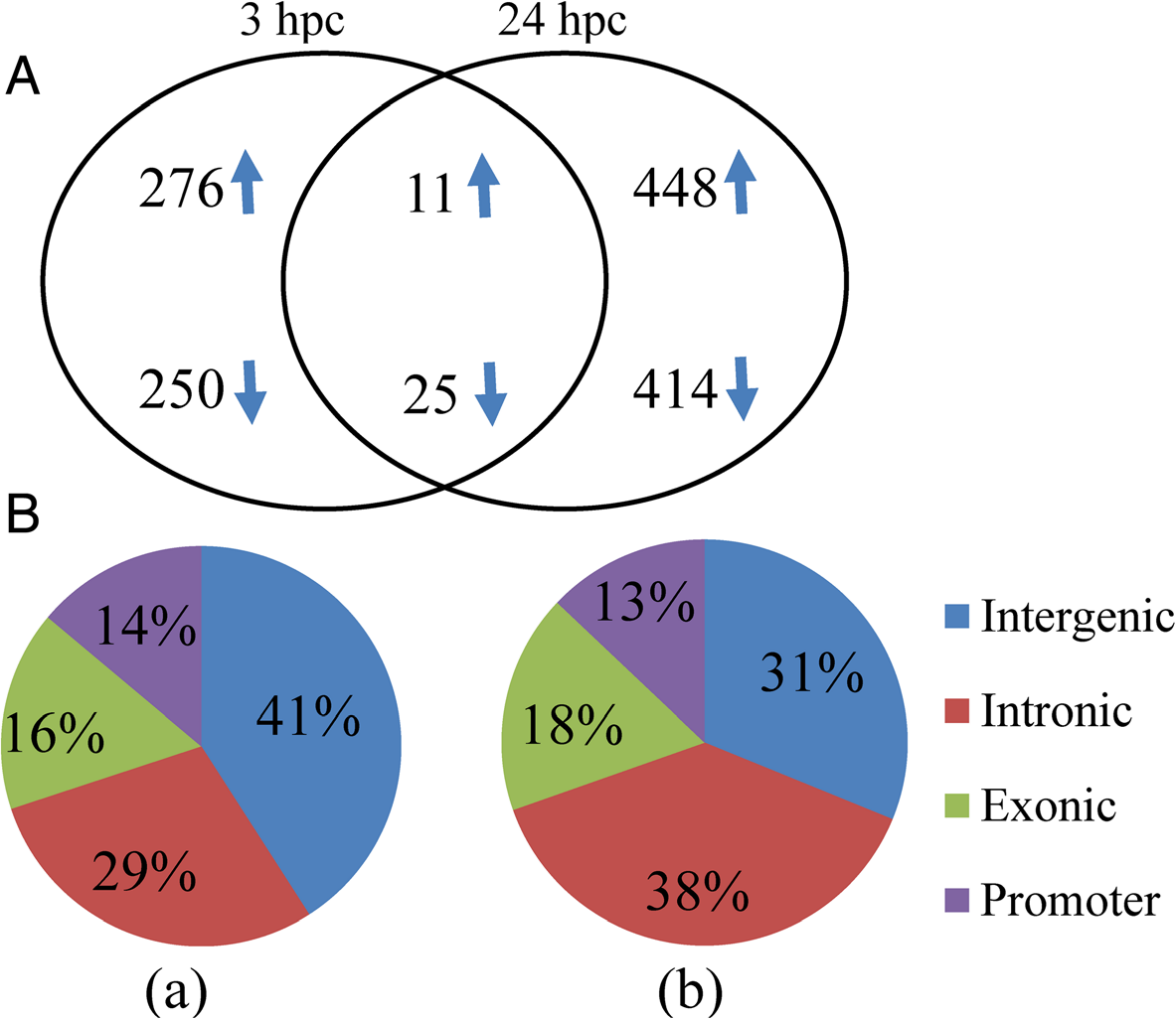
Significant differentially methylated CpGs (DMC) at *E. coli* 3hpc and *E. coli* 24hpc compared to control (**a**). The distribution of DMC at *E. coli* 3hpc (a) and *E. coli* 24hpc (b) in different genomic features (**b**)

**Fig. 2:**
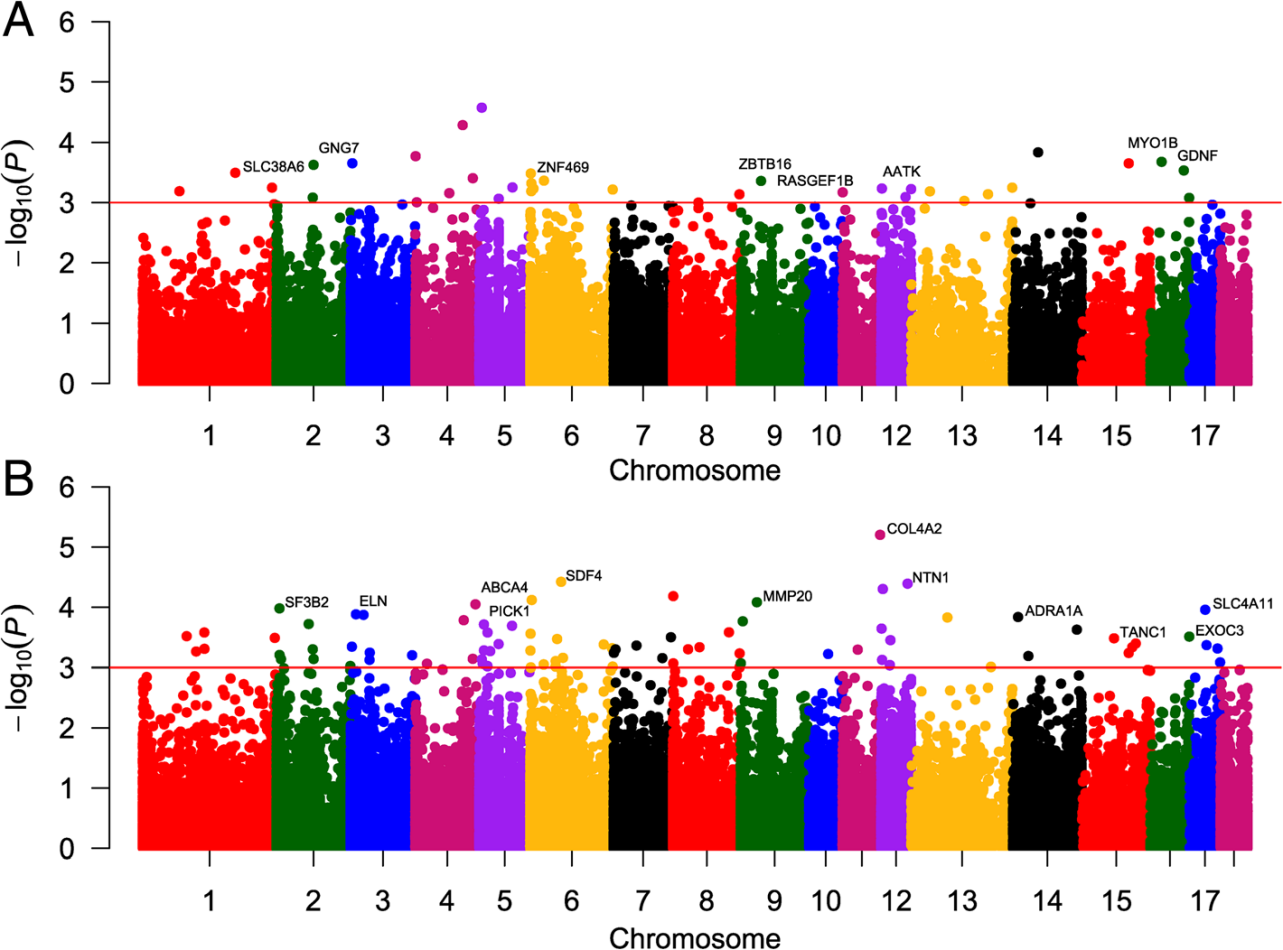
The distribution of differentially methylated CpG loci across the chromosomes is shown as Manhattan plots for *E. coli* 3hpc vs control (**a**) and *E. coli* 24hpc vs control (**b**). Each point represents a CpG site, with genomic position on the x-axis and –log10 of the *p-value* for differential methylation between treatment group and control on the y-axis. The red horizontal line indicates the genome-wide significance threshold of *P* < 0.001

**Fig. 3:**
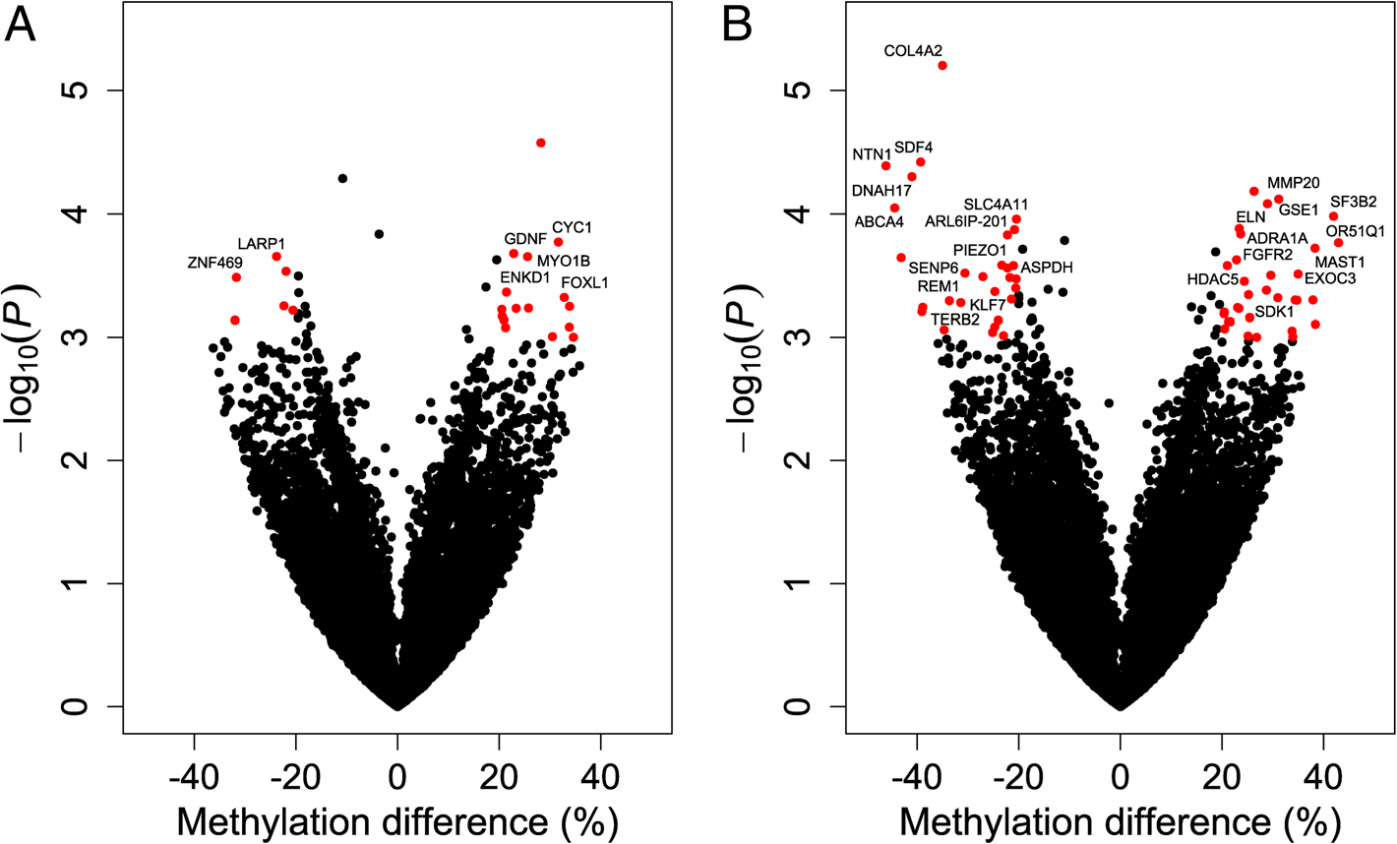
Representation of significant DMC using volcano plots for *E. coli* 3hpc vs control (**a**) and *E. coli* 24hpc vs control (**b**). Differences in mean methylation percentages between the control and *E. coli* challenged groups were plotted on the x-axis. The y-axis represents the negative *P value* for their association. The DMC mapped to the genes are marked in blue

The Venn diagram shown in Fig. 1a indicates the numbers of differentially methylated loci at both comparing (3 vs control and 24 hpc vs control). The proportion of these loci present in different genomic regions (promoter, exon, intron or intergenic) is shown in Fig. 1b. Manhattan plots were generated to show the chromosome-wide distribution of differentially methylated CpG sites identified in this study (Fig. 2). Statistically significant CpG sites are indicated as being above the cut off negative *P* values (3) on the log 10 scale. The chromosome wide distributions of CpG sites for the comparisons groups (3 hpc vs. control, 24 hpc vs. control) were shown (Fig. 2a & b). Similarly, the volcano plots depicted for the significant CpG sites based on both *P* values and methylation differences of 20% between *E. coli* challenged and control PMEC the groups (3 hpc vs. control, 24 hpc vs. control) (Fig. 3a & b).

**Fig. 4:**
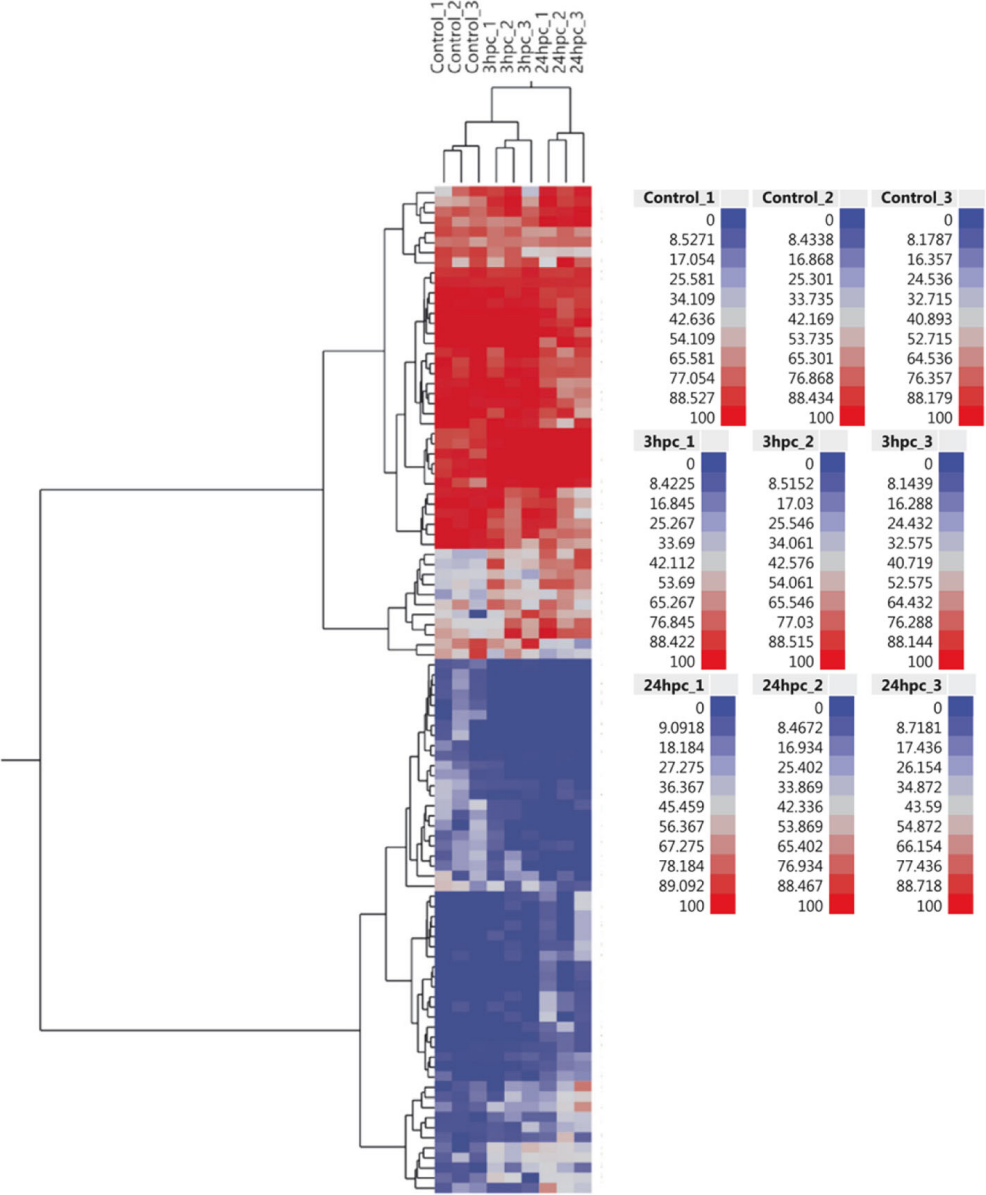
Heat map and hierarchical cluster analysis of the top 100 differentially methylated CpG identified in the study. The red colour in the heatmap indicates hypermethylated loci and the blue indicates hypomethylated loci. Hierarchical clustering dendrogram reveals distinct differential methylation patterns among different groups

**Fig. 5:**
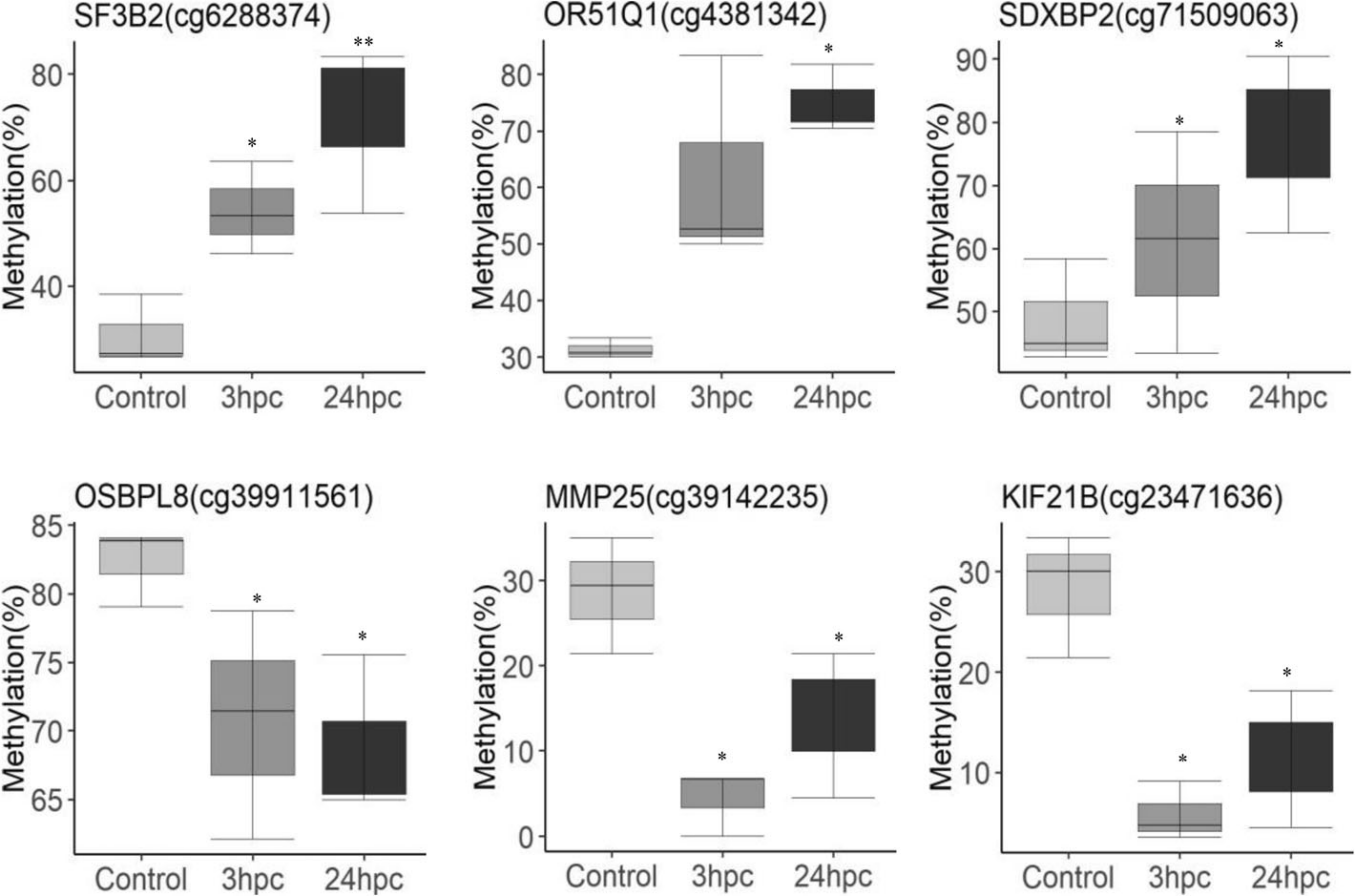
Differentially methylated CpG that were mapped to the genes and compared between control, *E. coli* 3hpc and *E. coli* 24hpc. The indicated significant differences (** *P < 0.001* and * *P < 0.05*) were observed between the control and E. coli challenged samples

The three sample groups of PMEC (Control, *E. coli* 3 hpc & *E. coli* 24 hpc) were examined by cluster analysis using hierarchical clustering method. The heatmap shows differential methylation of the top 100 CpG sites between different groups (Fig. 4). The top 10 significant CpG sites possibly differentially methylated at the in early phase of infection were selected by pair-wise comparison between *E. coli* 3 hpc and control samples. These differentially methylated mapped to genes including *CYC1* (cg605583), *GDNF* (cg22964888), *PTHR20855_SF21* (cg3759161), *MYO1B* (cg95955381), *GNG7* (cg76083105) and *LARP1.* Similarly, the top ten differentially methylated loci at the late phase of infection were identified by pairwise comparison of *E. coli* 24 hpc and control samples. These differentially methylated loci mapped to genes including *CYB5R1* (cg24937951), *COLA4* (cg77052099), *SDF4* (cg63545568), *NTN1* (cg54274409), *DNAH17* (cg3518148), *GSE1* (cg3323937), *MMP20* (cg33280374), *ABCA4* (cg123320105) and *SF3B2* (cg6288374) (Table 2). Figure 5 displays representative differentially methylated loci and their associated genes that exhibited similar methylation patterns (increased or decreased methylation) at both treatment time points (3 hpc and 24 hpc).

**Table 2:** Top 10 differentially methylated CpG sites and their associated genes in *E*. *coli 3 hpc and E. coli* 24 hpc compared to control

CpG site	Chr	Strand	Gene name	*P* value	Distance to gene feature (TSS ± bp)	Islands/shore
*E. coli* vs Control						
cg5322871	5	+	EFCAB6	2.64E-05	− 58243	
cg97042458	4	–	FLG2	5.15E-05	59383	cpg island
cg51707353	14	–		1.46E-04	− 16765	
cg605583	4	–	*CYC1*	1.69E-04	3413	
cg22964888	16	+	*GDNF*	2.10E-04	931	cpg island
cg3759161	3	–	*SF21*	2.22E-04	16920	
cg95955381	15	+	*MYO1B*	2.23E-04	47142	
cg76083105	2	–	*GNG7*	2.36E-04	28337	
cg68450973	16	–	*LARP1*	2.92E-04	33945	cpg island
cg190005969	1	–	SLC38A6	3.20E-04	81855	
*E. coli* vs Control						
cg24937951	10	+	*CYB5R1*	1.97E-10	− 6265	
cg77052099	11	+	*COL4A2*	6.26E-06	54975	
cg63545568	6	–	*SDF4*	3.78E-05	− 782	
cg54274409	12	–	*NTN1*	4.06E-05	− 32552	cpg shore
cg3518148	12	–	*DNAH17*	4.98E-05	32968	cpg island
cg727575	8	–		6.54E-05	68814	cpg island
cg3323937	6	–	*GSE1*	7.58E-05	26142	
cg33280374	9	+	*MMP20*	8.26E-05	27673	
cg123320105	4	+	*ABCA4*	8.94E-05	− 51088	
cg6288374	2	–	*SF3B2*	1.04E-04	5221	cpg island

### Functional analysis of differentially methylated genes

The annotated genes associated with differentially methylated CpG sites were submitted to functional analysis. This revealed the molecular functions, cellular processes, and biological disorders associated with genes identified during the early (3 hpc) and late (24 hpc) phases of *E. coli* challenge. The top 4 molecular and cellular functions at 3 hpc were cellular function and maintenances, cell death & survival, cell morphology, cellular assembly and compromise. Similar functions were found at 24 hpc in addition to post-translation modification, cellular growth, movement and proliferation. The diseases most associated with these genes were inflammatory diseases, organismal injury & abnormalities, and immunological diseases (Table 3). By comparing 3 hpc and 24 hpc groups, the top 4 molecular and cellular functions were cellular compromise, cell morphology, cell cycle and

**Table 3:** Summary of top diseases, molecular & biological functions associated with the differentially methylated genes in *E. coli* 3hpc and *E. coli* 24hpc

	*P* value	No. of molecules
*E. coli* 3 hpc vs Control		
Diseases & disorders		
Inflammatory disease	2.33E-02 - 3.80E-05	20
Organismal injury and abnormalities	2.33E-02 - 3.80E-05	89
Cancer	2.33E-02 - 5.98E-04	67
Endocrine system disorders	2.33E-02 - 5.98E-04	14
Molecular & cellular functions		
Cellular function and maintenance	2.33E-02 - 6.07E-05	32
Cell death and survival	2.33E-02 - 7.08E-05	46
Cell morphology	2.33E-02 - 1.81E-04	21
Cellular assembly and organization	2.33E-02 - 1.81E-04	35
Physiological system development & function		
Cardiovascular system development & function	2.33E-02 - 6.07E-05	22
Tissue development	2.33E-02 - 6.07E-05	35
Reproductive system development & function	2.33E-02 - 5.98E-04	21
Organismal development	2.33E-02 - 6.43E-04	48
*E. coli* 24 hpc vs Control		
Diseases & disorders		
Organismal injury & abnormalities	2.12E-03 - 3.79E-08	157
Cancer	2.12E-03 - 3.79E-08	129
Haematological diseases	2.12E-03 - 1.40E-07	58
Immunological diseases	1.80E-03 - 4.47E-07	52
Molecular & cellular functions		
Cell death & survival	1.80E-03 - 1.06E-06	90
Post-translation modification	1.09E-03 - 4.90E-06	36
Cellular development	2.07E-03 - 6.72E-06	57
Cellular growth & proliferation	2.07E-03 - 6.72E-06	46
Physiological system development & function		
Organismal survival	6.30E-04 - 7.56E-11	95
Cardiovascular system development & function	2.11E-03 - 1.08E-08	66
Organismal development	2.11E-03 - 1.08E-08	105
Embryonic development	2.07E-03 - 1.71E-07	79
*E. coli* 3 vs 24 hpc		
Diseases & disorders		
Cancer	4.41E-02 - 2.18E-06	136
Organismal injury & abnormalities	4.59E-02 - 2.18E-06	137
Gastrointestinal diseases	4.41E-02 - 3.52E-06	120
Developmental disorder	4.59E-02 - 6.03E-04	24
Molecular & cellular functions		
Cellular compromise	3.96E-02 - 4.05E-04	9
Cell morphology	4.41E-02 - 1.43E-03	24
Cell cycle	2.55E-02 - 1.86E-03	8
Cell-To-Cell signaling and interaction	4.41E-02 - 2.17E-03	17
Physiological system development & function		
Connective tissue development and function	4.82E-02 - 1.86E-03	12
Reproductive system development and function	4.88E-02 - 2.17E-03	19
Digestive system development and function	3.79E-02 - 2.59E-03	6
Organ morphology	4.41E-02 - 2.59E-03	17

cell-to-cell signaling and interaction. No inflammatory diseases or immunological diseases genes was found by comparing 3 hpc and 24 hpc groups. Further, differentially methylated CpGs that are located within the promoter regions (TSS ± 2000 bp) were grouped by k-means clustering and the affected top biological functions were identified. The innate immune regulation and stress activated mechanisms were identified in cluster groups at 3 h and 24 h respectively (Additional file 3: Figure S3). Additional k-means clusters of the union of differentially methylated CpGs of all three groups the most important biological functions of the group were performed (Additional file 4: Figure S4).

The most enriched transcription factors were identified by considering genes with differentially methylated CpGs within their promoter regions and using DAVID with the *Homo sapiens* UCSC TFBS function, a function not yet supported in *Sus scrofa spp. the* results revealed the enriched transcription factors identified by comparing *E. coli* 3 hpc to control samples (Table 4). One such transcription factor, *PAX5*, is known to influence B-cell differentiation and trafficking by regulating large numbers of downstream genes involved in immune functions. Other enriched factors with interesting roles in immune response include *MSX1* (innate immunity), *CREB* (cAMP signalling), and *IRF2* (Interferon Regulatory factor-2). Similarly, *E. coli* 24 hpc vs unchallenged analysis revealed enrichment of transcription factors (Table 4). Among these *PAX5, CREB*, and *AP4* were again found to be enriched along with other transcription factors such as *XBP1* and *E2F* that are involved in immune response and other biological functions.

**Table 4:** List of transcription factors enriched in differentially methylated gene in *E. coli* 3hpc and *E. coli* 24hpc

*E. coli* 3 hpc vs Control				
	Genes	Fold enrichment	*P-Value*	FDR
Transcription factor				
*PAX5*	62	1.5	8.20E-06	1.40E-03
*USF*	62	1.4	3.60E-05	3.10E-03
*HOX13*	51	1.6	8.00E-05	4.70E-03
*HMX1*	51	1.6	9.00E-05	4.00E-03
*BACH1*	54	1.5	1.30E-04	4.70E-03
*AP4*	64	1.4	2.20E-04	6.50E-03
*LUN1*	53	1.5	2.30E-04	5.90E-03
*MSX1*	45	1.5	8.90E-04	1.10E-02
*CREB*	46	1.5	9.90E-04	1.00E-02
*IRF2*	44	1.5	1.50E-03	1.30E-02
*E. coli* 24 hpc vs Control				
*LMO2COM*	102	1.4	2.20E-06	3.90E-04
*OLF1*	81	1.5	2.80E-05	2.40E-03
*MYOD*	93	1.3	8.10E-05	4.80E-03
*AP4*	104	1.3	9.40E-05	4.10E-03
*PAX5*	91	1.3	7.00E-04	2.00E-02
*E2F*	91	1.3	1.00E-03	2.60E-02
*MSX1*	69	1.4	2.00E-03	2.50E-02
*CREB*	87	1.3	1.30E-03	2.80E-02
*HEN1*	92	1.3	1.50E-03	2.90E-02
*XBP1*	77	1.3	7.60E-03	6.50E-02

### Pyrosequencing analysis

**Fig. 6:**
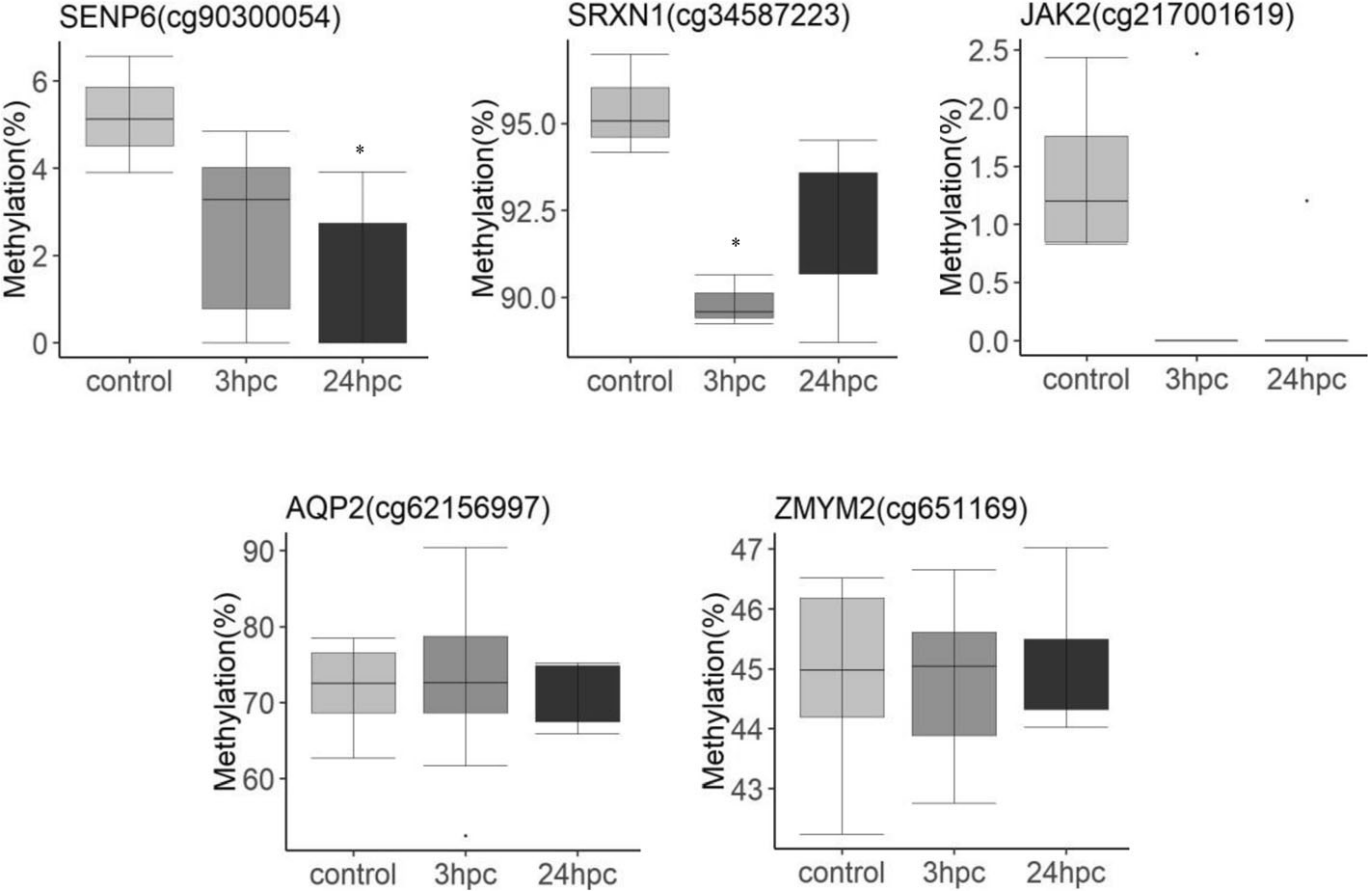
Pyrosequencing validation of CpG sites identified in the genome wide methylation including *SENP6* (cg90300054), *SDF4* (cg63545568), *JAK2* (cg217001619), *SRXN1* (cg34587223), *ZMYM2* (c651169). Methylation percentages were plotted for control, *E. coli* challenged (3hpc and 24hpc). The data represents full range of variations (from min to max), the likely range of variation (IQR) and median value. The indicated significant differences (*P < 0.05*) were observed between the control and *E. coli* challenged samples

The genome wide analysis identified differentially methylated CpG sites mapped to different genes. Based on these results, *SENP6* (cg90300054), *SRXN1* (cg34587223), *JAK2* (cg217001619), *AQP2* (cg62156997) and *ZMYM2* (651169) were selected for bisulfite PCR and pyrosequencing analysis. The results validated the methylation patterns of CpG sites for *SRXN1*, *SENP6*, and *JAK2* that were hypomethylated in *E. coli* after 24 h challenged cells compared to the control cells. However, the pyrosequencing validations were not significant for CpG sites in *AQP2*, and *ZMYM2* genes (Fig. 6 and Additional file 5: Figure S5).

### Expression analysis using quantitative RT-PCR

**Fig. 7:**
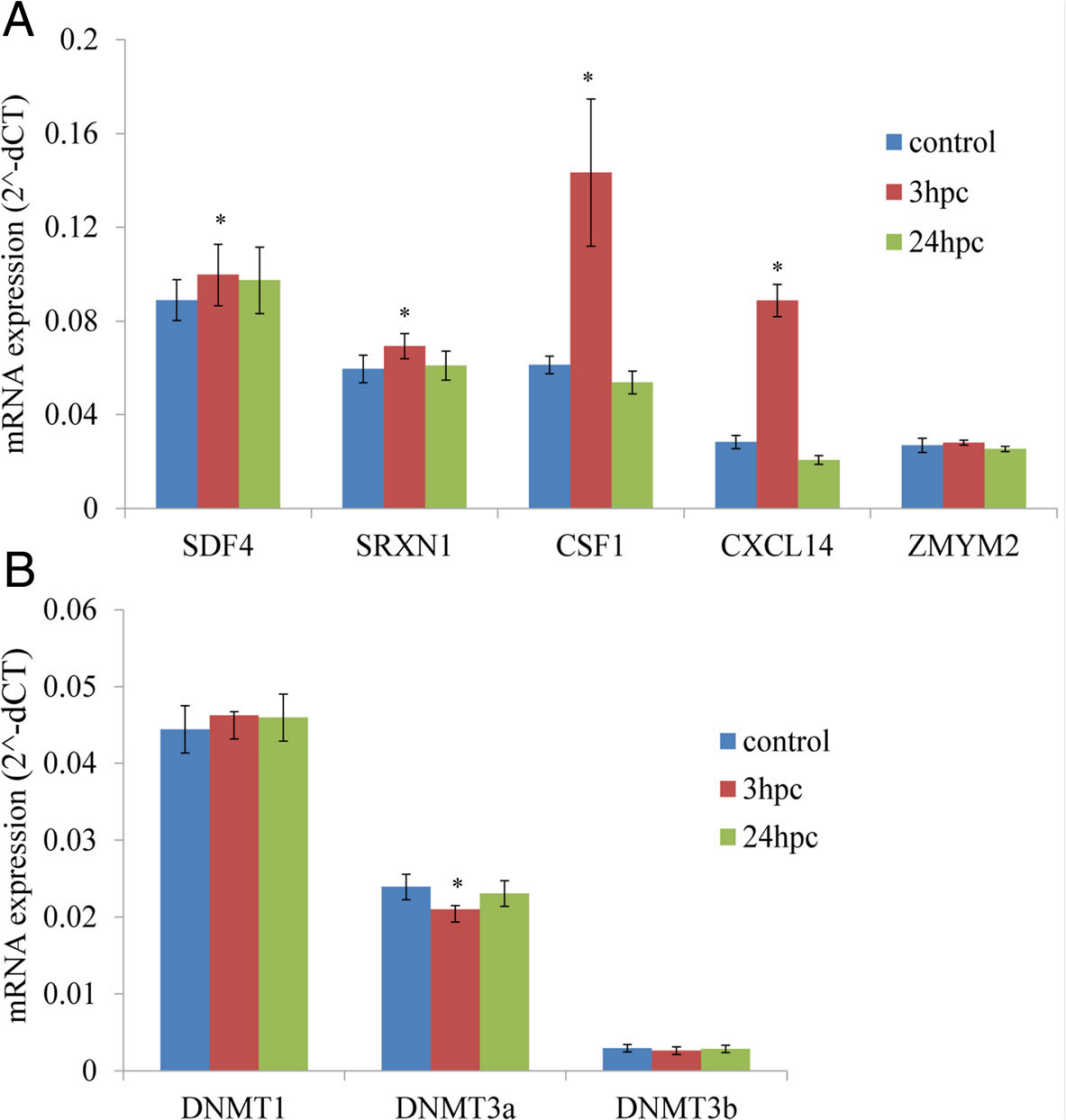
Gene expression levels analysed using quantitative mRNA expression analysis. Differential expression patterns of selected genes that had significant hypomethylation in their upstream regulatory regions around TSS (**a**). Expression patterns of different DNA methyl transferase (DNMT) enzymes (**b**). A significant difference (*P < 0.05*) was indicated

The differentially methylated CpG loci within promoter or regulatory regions were deemed quite likely to influence the expression of their downstream genes. We selected genes with the significant hypomethylated CpG sites in the promoter (− 2 kb to + 1 kb from TSS) as identified in our study for quantitative expression analysis. Significantly higher expression was noticed for stromal derived factor 4 (*SDF4*), sulforedoxin (*SRXN1*), Colony stimulating factor 1 *(CSF1*), and *CXCL14*. The zinc finger transcription factor (*ZMYM2*) gene exhibited no change in expression between when comparing control and *E. coli* challenged PMEC (Fig. 7). The expression of DNA methyl transferases (*DNMTs*) was also examined. There was a significant reduction in the expression of *DNMT3a* (de novo methyl transferase) in 3 hpc cells, however no major changes were observed for other related genes (*DNMT1* and *DNMT3b*).

## Discussion

*E. coli* infection is the main source of mastitis in domestic animals, and epigenetic mechanisms have a strongly influence on the expression of genes during *E. coli* and *Staphylococcus aureus* (*S. aureus*) associated mastitis [13–15]. The LPS endotoxin, a major *E. coli*-derived pathogenic factor for inflammatory response in mastitis, induces changes in innate immunity genes in dairy cows, and a role for DNA methylation has been demonstrated for this process [16]. Exposure of bovine dermal fibroblasts to a demethylating agent, 5-aza-2′-deoxycitidine (AZA), altered their response to an LPS challenge, indicating the role of DNA methylation in immune response [17]. Also, a recent genome wide methylation analysis revealed DNA methylation differences contributing to LPS-induced immune response in bovine fibroblasts [18].

Pigs are an important disease model of coliform mastitis, and the present study is the first to investigate the underlying epigenetic mechanisms mediating host-pathogen interaction in cultured porcine mammary cells. To achieve this, we have taken advantage of the clear differences in gene expression patterns occurring during the immediate (3 h) and late immune response (24 h) observed in our earlier studies [11, 12] where we demonstrated that pathogenic *E. coli* challenge of PMEC resulted in up-regulation of immune response genes, such as chemokines, cytokines, and cell adhesion molecules.

In the current study, we examined changes in DNA methylation induced by *E. coli* challenge. The genome wide methylation patterns indicated comparatively lower CpG methylation in upstream promoter regions compared to gene body regions, and a bimodal distribution of CpG methylation levels was observed. These findings are consistent with prior studies on genome wide CpG methylation in pig [19, 20]. Our analysis revealed 561 and 898 differentially methylated CpG loci associated with the immediate and late responses to an *E. coli* challenge, respectively at *P <* 0.01. Annotation of these CpG probes identified differentially methylated genes, and pathway analysis revealed that the highest enriched functional associations of these genes were inflammatory diseases and organismal injury. In terms of molecular and cellular functions, the top differentially methylated probes associated with cell death & survival, post-translation modifications, cell morphology, cell growth & proliferations. Further differentially methylated CpG’s were clustered into different groups of related genes that indicated immune response as one of the major biological process affected including other cellular processes. Promoter regions are known to have binding sites for different transcription factors that drive gene expression. The genes with differentially methylated CpG’s in their promoters were used to identify relevant transcription factors, and several such identified factors have known prominent roles in inflammation, including *PAX5, AP4, CREB, IRF2* and *XBP1. PAX5* is a transcription factor that regulates various B cell functions including activation of NF-kB [21]. *IRF2* (Interferon regulatory factor 2) regulates *IFN-β* expression and was found to inhibit LPS induced proinflammatory responses [22]. *CREB* (cAMP-responsive element binding protein) transcriptionally activates pro-inflammatory genes, such as *IL-2, IL-6*, and *TNF-α* by binding to the cAMP response element (CRE) in their promoter sequences [23]. *XBP1* (X-box binding protein 1) is a positive regulator of *TLR* gene induction and plays a major role in interlinking LPS-associated *TLR* activation to Endoplasmic Reticulum (ER) stress [24].

In general, it is known that hypomethylation of CpG motifs in the promoter regions of genes enhances their transcription. As shown in earlier studies, infection induced hypomethylation of CpGs leads to higher expression of immune genes [7, 25]. In our study, we examined the expression of *SDF4, SRXN1, CSF1, CXCL14* and *ZMYM2*, all genes which we found to have a significant challenge-dependent reduction in CpG methylation in their upstream regulatory regions around TSS. *SDF4, SRXN1,* and *CXCL14* were selected from hypomethylation CpGs at 24 h after *E. coli*-challenged, while *CSF1* were selected from hypomethylation CpGs at 3 h after *E. coli*-challenged. The quantitative transcript estimation showed that only the expression of *SDF4* and *CSF1* in *E.coli*-challenged cells at 24 h and 3 h was significantly higher than in control cells. However expression pattern and methylation level of *SRXN1,* and *CXCL14* didn’t show a negative relationship. Many studies reported different methylation sites associated with expression, regardless of the directional change in expression and methylation level [26]. This may because DNA methylation is not exclusively associated with repression of transcription initiation [27]. The expression of *ZMYM2* was also higher in the challenged cells, although this difference was not significant. These results confirmed that the CpG methylation in the promoter regions of these genes correlates with changes in gene expression.

The present study revealed promoter hypomethylation of *SDF4* and significantly higher expression of *SDF4* in *E.coli*-challenged PMEC. SDF (stromal derived factors) belong to the CXC subfamily of proteins, are expressed in different tissues, and function as chemokines to attract inflammatory cells [28]. Increased levels of *SDF1* (CXCL12) can be seen in inflammatory conditions, such as sub-acromial bursitis and acute liver injury [29, 30]. *SDF4* expression was found to be significantly reduced in mammary tumours compared to normal tissue and low levels of *SDF4* are linked to a poor prognosis [31]. Our results indicate a possible epigenetic mechanism for regulating *SDF* expression and immune response in mammary tissues.

In this study, the upstream regulatory region of the *SRXN1* gene had reduced CpG methylation and significantly increased expression in *E. coli*-challenged cells. Sulfiredoxin (*SRXN1*) is an antioxidant enzyme that prevents ROS injury to cells, can reduces oxidized cysteine residues of peroxiredoxin proteins (Prx I- IV), and facilitate removal of free radicals [32]. LPS released form *E. coli* induces reactive oxygen species (ROS) formation leading to upregulation of antioxidant enzymes, including SRXN1, and SRXN1 production in macrophages was shown to protect mice from LPS induced endotoxic shock [33]. Earlier studies indicate that the promoter region of the *SRXN1* gene has putative *NF-kB* and *AP1* transcription factor binding sites required for up-regulation of *SRXN1* expression by LPS treatment [34]. Hypomethylation may enhance the binding of these transcription factors at the promoter region to drive the *SRXN1* expression.

In the present study, we found increased expression of *CSF1* in the immediate early phase of *E.coli* challenge but not at the later phase. Colony stimulating factor-1 (*CSF-1*) has been shown to differentiate peripheral blood monocytes into tissue macrophages [35, 36]. *CSF-1* expression is induced by uropathogenic *E.coli* infection and has a critical role in bacterial clearance during infection [37, 38]. The higher expression of *CSF1* corresponds to hypomethylation in the upstream regulatory regions of the *CSF1* gene. Curiously, expression of the *CSF1* receptor (CSF1R) is also known to be regulated by DNA methylation of its promoter region [39]. *CXCL14* is an additional gene found to be regulated in this study. CXCL14 is a chemokine that plays inflammatory modulator and host defence roles in epithelial tissues [40, 41]. It was shown to have antimicrobial activity in the respiratory tract and contributed to the clearance of *Streptococcus pneumoniae* [42]. There is evidence for epigenetic regulation of *CXCL14* in prostate cancer cells. Treatment of these cells with the demethylating agent 5-aza-2-deoxycytidine affects a hypermethylated CpG island in the *CXCL14* gene promoter resulting in the recovery of CXCL14 expression and restoration of chemotaxis [43]. Methylation-mediated control of *CXCL14* can also be inferred from our study where an *E.coli* challenge induced hypomethylation in the promoter region of the *CXCL14* gene associated with higher levels of expression. These results indicate the potential involvement of epigenetic mechanisms in regulating host cell response to *E. coli* infection.

Some of the genomic regions showed different methylation between infection groups, including CpGs at SSC5 39.78–40.07 Mb (OSBPL8) and at SSC9 63.38–63.39 Mb (MROH9). These regions (9:63272406–63401079 bp and 5: 39774063–39828561 bp) contained large CpG islands (CGI) (genome order: Sscrofa11.1). These regions also showed heterogeneity in methylation and changes in the degree of methylation between infection groups. But both of these regions are far away from the promoter site of the transcripts.

DNA methyltransferases (DNMT) are the principle enzymes responsible for controlling epigenetic modifications, and DNMT3a and DNMT3b are responsible for de novo DNA methylation. In the present study, the quantitative expression results indicated significantly reduced expression of *DNMT3a* in the *E. coli* infected PMEC compared to the control group; however, no changes in the patterns of either *DNMT1* or *DNMT3B* were observed. Hypomethylation in upstream regions of immunity genes may partially be explained by the reduced expression of *DNMT3a*. It was previously shown that UPEC infections increase *DNMT1* expression, the enzyme responsible for maintenance DNA methylation [5]. The level of various DNA methyl transferase enzymes is likely to modulate the expression of many genes during bacterial infection.

## Conclusion

In conclusion, the present study identified first time genome wide differential CpG methylation patterns induced by *E. coli* challenge in PMEC. CpG methylation changes in the upstream regulatory regions were used to identify enriched transcription factors that regulate immune response pathways. Further, reduced DNA CpG methylation was observed in the immune response genes with corresponding increases in their expression. These results indicate potential epigenetic mechanisms that regulate inflammation during coliform mastitis in pigs.

## Methods

### Primary culture of PMEC

German Law of Animal Protection guidelines were followed for collecting the tissues. Animal Care Committee at Leibniz Institute of Farm Animal Biology (FBN), Dummerstorf 18196, Germany approved the experiments. The sows were weighed and slaughtered by electronarcosis followed by exsanguination in the experimental abattoir of the FBN. Tissues from mammary complexes cranial of the navel were collected aseptically immediately after slaughter from each individual. After tissue collection all animals underwent routine processes of the slaughterhouse. Veterinary inspection of the animals before slaughtering and of their carcasses and organs after slaughter proofed that they were without any impairment, disease symptoms and pathological signs. Primary cultures of PMEC were obtained as described in our earlier studies [11]. Briefly, tissues were isolated from mammary glands of three lactating sows, minced and digested with collagenase enzyme. The cells were washed and suspended in complete growth medium. Primary cultures of PMECs were established by removing other cell types, such as fibroblasts and adipocytes, by selective trypsinization (Trypsin/EDTA-0.25/0.02%, Sigma-Aldrich). The fibroblasts and adipocytes detach more rapidly; whereas, epithelial cell islands remain adhered to the surface of the culture flasks. The procedure was repeated several times until a uniform and confluent monolayer of epithelial cells was obtained.

### *E. coli* challenge to the cultured PMEC

The present study uses the same *E. coli* strain (gMEc240, sequence type 101, phylogroup B1, C+) isolated from milk of PDS-positive sows described in our previous study [11, 12]. Briefly, approximately 4.4 × 10^5^ PMEC from each 3 sow (3 biological replicates) were seeded and cultured in collagen-coated 6-well plates (1:10 collagen R in distilled water, Menal, Emmendingen, Germany) in complete medium without APS (three technical replicates per individual and treatment condition). After 24 h, the medium was changed. Forty-eight hours after seeding, the cells reached ~ 90% confluency. Then, PMEC were challenged with 10^7^/ml heat-inactivated *E. coli* for 3 h or for 24 h. The control PMEC cells were not challenged with *E. coli.* The medium was discarded, and the cells washed three times with phosphate buffered saline (PBS, PAA) to remove bacteria after incubation periods. The experiments included in triplicates for each three animals in three groups (control, 3 h and 24 h). In total 27 genomic DNA and total RNA samples were isolated from treated and control PMEC.

### Preparation of RRBS libraries

Equivalent amount of genomic DNA from three technical replicates per individual animal were pooled. In total 9 pooled samples of DNA, three for each *E. coli* challenged PMEC at two different time points (3 h post-challenge (hpc) and 24 hpc) and unchallenged control. PMEC were used for libraries construction. RRBS libraries were prepared using 2 μg of pooled genomic DNA with a 1% spike-in control (unmethylated cl857 Sam Lambda DNA (Promega)). The genomic DNA was digested with Msp I and TaqαI. Double-enzyme (MspI and TaqαI) digestion RRBS with increased size-selected fragments will enhance genome-wide CpG coverage. The digested fragments were end repaired, A-tailed and ligated with the C-methylated adaptor sequences TruSeq Nano DNA Sample Preparation kit (Illumina) by following the manufacturer’s protocol (Illumina, San Diego, CA). The DNA fragments were later size selected for 40–200 bp with a 2.5% NuSieve 3:1 agarose gel and extracted using the Zyomclean™ Gel DNA Recovery Kit (Zymo Research). The purified DNA was treated with bisulfite using the EZ DNA Methylation-Gold Kit™ (Zymo Research). The preparative scale PCR was performed for 15 cycles and PCR products were purified with the DNA Clean and Concentrator Kit™ (Zymo Research). The qualities of the RRBS libraries were assessed using an Agilent DNA 1000 kit (Agilent Technologies). NGS of the 9 RRBS libraries were performed on an Illumina HiSeq2500 for single-reads of 114 bp at the FBN, Dummerstorf. The bcl2fastq2 conversion software v2.19 was used to convert base call (BCL) files from a sequencing run into FASTQ files that were used for further analysis.

### Bioinformatics analysis

The sequence reads were assessed for quality using FastQC and bases with Phred score greater than 20 were retained for further downstream analysis. RRBS introduces artificial CpG at the 3′ end of the fragments that are removed along with the adaptor sequences to avoid their inclusion in the methylation calling. The default settings for Trim Galore (v0.1.1.1, Babraham Bioinformatics, UK) were used for Illumina adaptor trimming as they specifically remove the first two bases from the 3′ end of the sequence such that the additional ‘C’ closest to the enzyme cut site is removed. The trimmed reads were mapped to the *in-silico* bisulfite converted porcine genome (11.1) using the Bismark alignment tool (v0.13.1, Babraham Bioinformatics, UK). Bisulfite treatment converts unmethylated cytosines to uracils whereas, methylated cytosine is not affected. The sequence reads were mapped to the pre-converted reference genome (Sscrofa 11.1), reads aligned to the multiple regions are removed and best uniquely mapped reads were used for methylation calling.

### Differential methylation analysis and annotation

Methyl call files from the Bismark aligner with the percent methylation score per base were taken as input files for analysis. The reads which cover all the treatment and control samples with a minimum coverage of 10 were only considered to increase the power of statistical test. The reads from the sex chromosomes, mitochondria, unannotated genome segments and those showing no methylation variation across all samples were filtered out. Differential methylation analysis was done using the MethylKit [44]. Logistic regression with “MN” basic overdispersion correction was applied for testing methylation proportion of each CpG between the treatment and control group samples. The methylation differences between the groups were considered to be statistically significant at *P <* 0.01. A heatmap was used to show the methylation differences between the groups using selected DNA methylation loci. The differentially methylated CpG were annotated to genomic features by using the genomation R/Bioconductor package.

### Bisulfite PCR and pyrosequencing

Differentially methylated CpGs identified by genome-wide analysis were validated using bisulfite PCR and pyrosequencing methods. The same genomic DNA which was used for genome wide methylation analysis was treated with bisulfite using EZ DNA Methylation-Gold Kit™ (Zymo Research). Primers were designed using the Pyrosequencing Assay Design Software (Additional file 9) and target region was amplified with PCR using AmpliTaq Gold DNA Polymerase (Applied Biosystems, Cat. No. 4311814). As most of the differentially methylated loci are in CpGislands, in addition to target CpGs, the additional adjacent CpGs were also included for pyrosequencing. Pyrosequencing was performed with PSQ™96MA as per the manufacturer’s instructions. The CpG methylation percentages were calculated using PyroMark CpG software 2.0 based on the height of the T and C peaks at the target CpG sites.

### Functional analysis

Functional network analysis was done to gain biological insights into top differentially methylated loci between *E. coli* challenged and control PMEC. Genes annotated from the selected CpG with *P* < 0.01 were included in the gene function network analysis. Ingenuity pathway analysis (Ingenuity Systems, Inc., CA, USA) with its core analysis features was used. Differentially methylated CpGs present between − 2.5 kb and + 1 kb from an annotated transcription start site (TSS) were considered as being in the promoter region of a gene. Such promoter gene IDs were used for transcription factor enrichment, and transcription factor binding site (TFBS) analysis was done using the default parameters of the UCSC_TFBS track of *Homo sapiens* due to the lack of a *Sus scrofa* data track and considering that most of the TFBS are conserved.

### Quantitative gene expression analysis

Expression analysis was done for selected genes with differentially methylated regulatory regions. RNA was isolated from 27 samples after the *E. coli* challenged and control PMEC using the TRI reagent (Sigma-Aldrich) following the manufacturer’s instructions. Isolated RNA was purified by the RNeasy Mini Kit (Qiagen) and DNase I treatment was done to remove the contaminating DNA. First strand cDNA was synthesized using SuperScript III MMLV reverse transcriptase (Invitrogen) with 1 μg of RNA, 500 ng oligo (dT) and 500 ng random hexamer primers (Promega). Quantitative real-time PCR was performed using the LightCycler® 480 Real-Time PCR System (Roche Diagnostics). *GAPDH* and *RPL32* were used as internal housekeeping control genes. The sequences of primers for the selected test genes and internal control genes were designed using Primer3 (v.0.4.1) (Additional file 9). The reaction conditions were: 95 °C for 5 min (initial denaturation), 45 cycles consisting of 60 °C for 15 s (denaturation), 72 °C for 25 s (extension/fluorescence acquisition). Melting curve analysis and agarose gel electrophoresis indicated single amplicon without any primer dimers. Threshold cycle values were normalized to the expression of the internal control genes, and significant differences in expression were assessed with ANOVA and *P* < 0.05 was considered statistically significant.

## Additional files

Additional file 1: **Figure S1.** Genomic distribution of mapped CpG residues from RRBS libraries to the known functional annotations of porcine RefSeq genes in-terms of reads fraction (A) and percentages (B). The genomic distribution of RRBS reads to the porcine genomic CpG island/CpG shore regions in terms of reads fraction (C) and percentages (D). (TIF 855 kb)

Additional file 2: **Figure S2.** Methylation levels of identified CpG sites. The bimodal distribution of CpG methylation was observed in all the samples (A). The methylation levels (%) at different genomic features such as CpG islands, CpG shores (B) and at Promotes, Exons, Introns and Intergenic regions (C) represented. (TIF 1017 kb)

Additional file 3: **Figure S3.** k-means clustering of differentially methylated genes (CpG in TSS ± 2000) with k = 2 and scaled as Z-score across rows. Top five gene ontology (GO) biological processes derived from each k-means clusters ranked based on the fold enrichment. A) E coli 3 hpc vs control, B) E coli 24 hpc vs control. (TIF 2836 kb)

Additional file 4: **Figure S4.** k-means clustering of differentially methylated common CpG that were present in both *E. coli* 3 hpc vs control and *E. coli* 24 hpc vs control and top five enriched biological biological process. (TIF 1258 kb)

Additional file 5: **Figure S5.** Differentially methylated CpG sites identified between *E. coli* 3hpc or 24hpc compared to the unchallenged control group from NGS data compare to pyrosequencing including SENP6 (cg90300054), SDF4 (cg63545568), JAK2 (cg217001619), SRXN1 (cg34587223), ZMYM2 (c651169). The y-axis for both box plots represents methylation level. Genes associated with the CpG are given. Box plot represents the range of variation and median value. (TIF 2466 kb)

Additional file 6: Differentially methylated CpG sites in *E. coli* 3hpc compared to the unchallenged control group. (XLSX 55 kb)

Additional file 7: Differentially methylated CpG sites in *E. coli* 24hpc compared to the unchallenged control group. (XLSX 75 kb)

Additional file 8: Differentially methylated CpG sites in *E. coli 3hpc* compared to *E. coli* 24hpc. (XLSX 71 kb)

Additional file 9: List of primers sequences used for quantitative gene expression and pyrosequencing. (DOCX 15 kb)

## Data Availability

All RRBS sequencing data have been deposited in the ArrayExpress database at EMBL-EBI (www.ebi.ac.uk/arrayexpress) under the accession number E-MTAB-7363.

## Abbreviations

LPS: Lipopolysaccharide
PDS: Postpartum dysagalactia syndrome
PMEC: Porcine mammary epithelial cells
RRBS: Reduced-representation bisulfite sequencing
