# Supplementary figures and images for: DNA methylation analysis of porcine mammary epithelial cells reveals differentially methylated loci associated with immune response against Escherichia coli challenge
Source: BMC Genomics. 2019 Jul 31;20:623. doi: 10.1186/s12864-019-5976-7 (PMC6670134; doi:10.1186/s12864-019-5976-7)

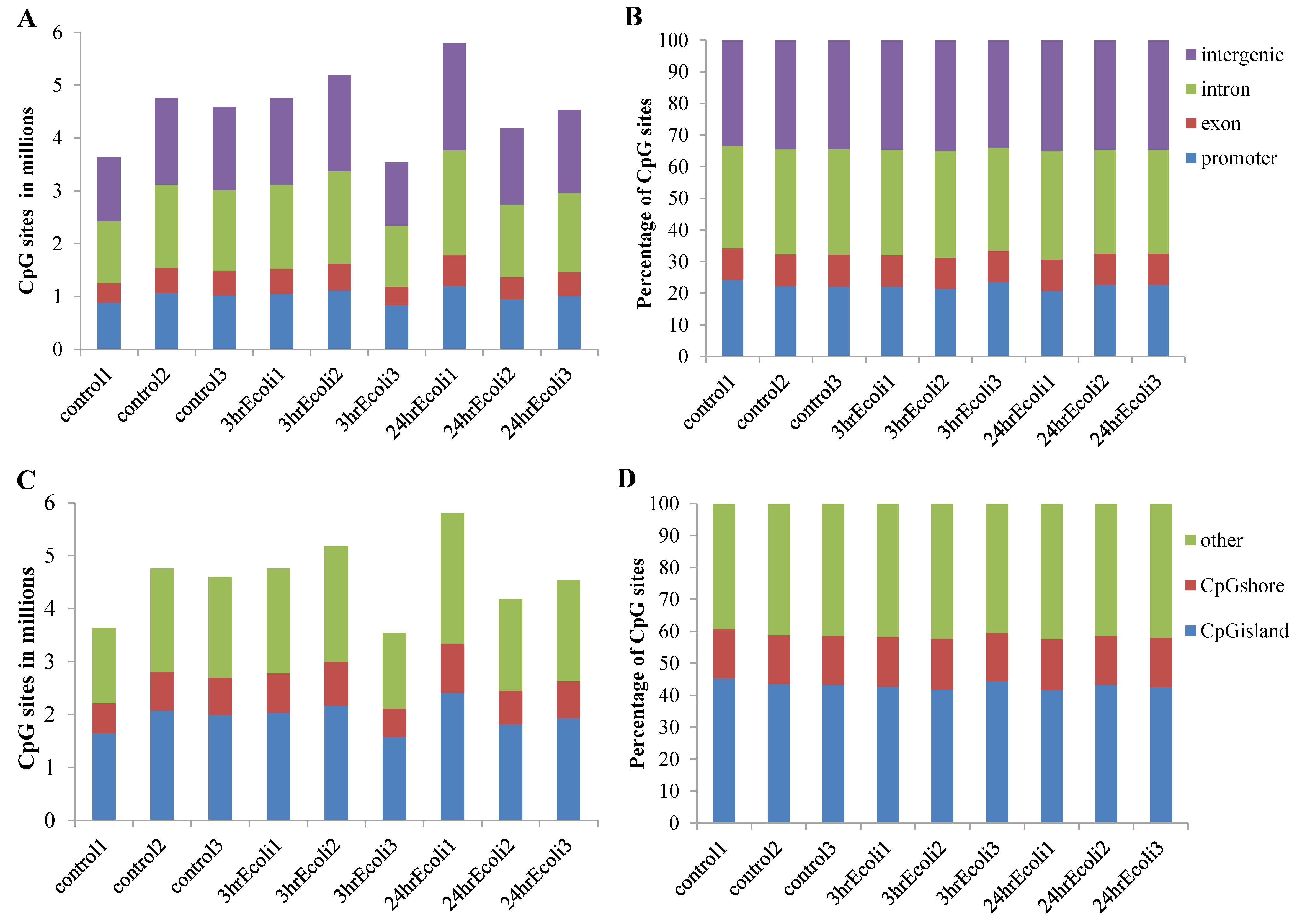

Supplement: Supplementary file 1 — Figure S1. Genomic distribution of mapped CpG residues from RRBS libraries to the known functional annotations of porcine RefSeq genes in-terms of reads fraction (A) and percentages (B). The genomic distribution of RRBS reads to the porcine genomic CpG island/CpG shore regions in terms of reads fraction (C) and percentages (D). (TIF 855 kb) [file 12864_2019_5976_MOESM1_ESM.tif]

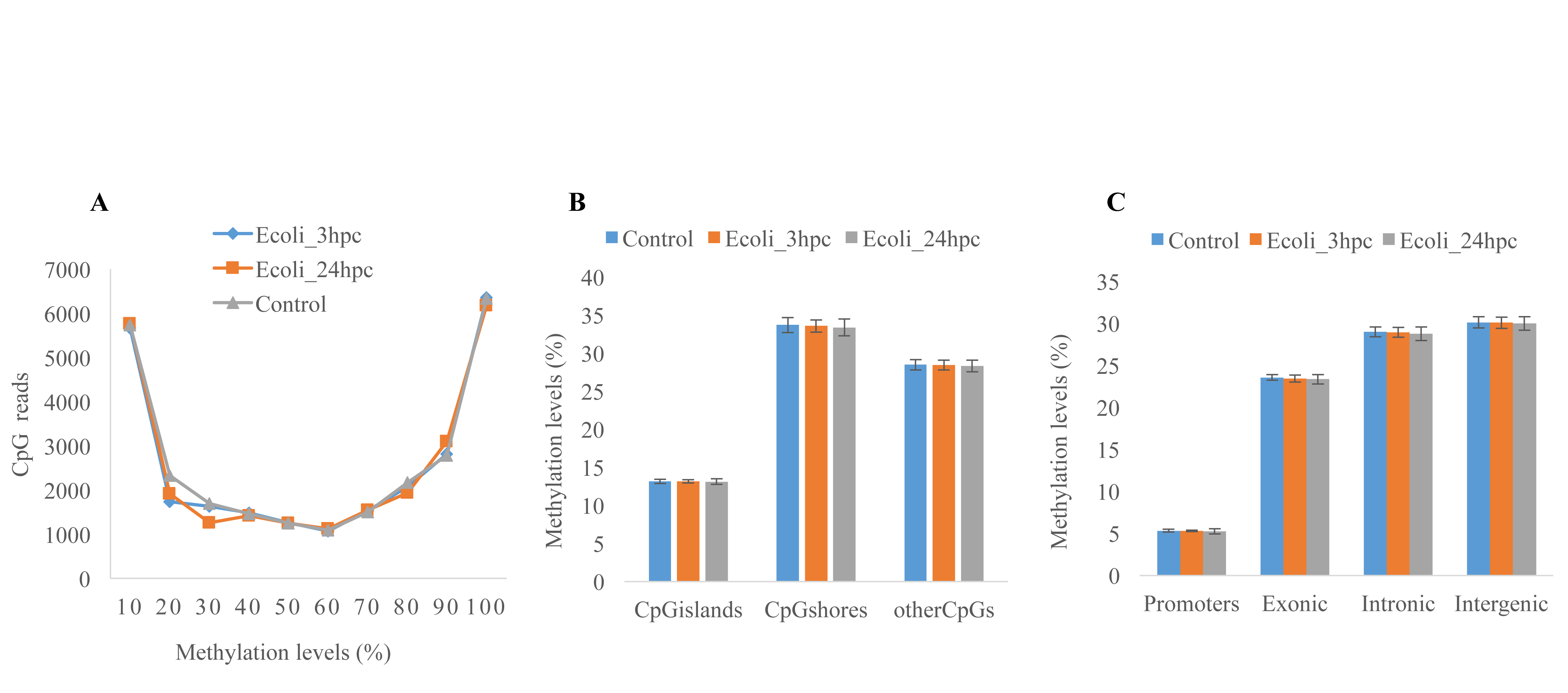

Supplement: Supplementary file 2 — Figure S2. Methylation levels of identified CpG sites. The bimodal distribution of CpG methylation was observed in all the samples (A). The methylation levels (%) at different genomic features such as CpG islands, CpG shores (B) and at Promotes, Exons, Introns and Intergenic regions (C) represented. (TIF 1017 kb) [file 12864_2019_5976_MOESM2_ESM.tif]

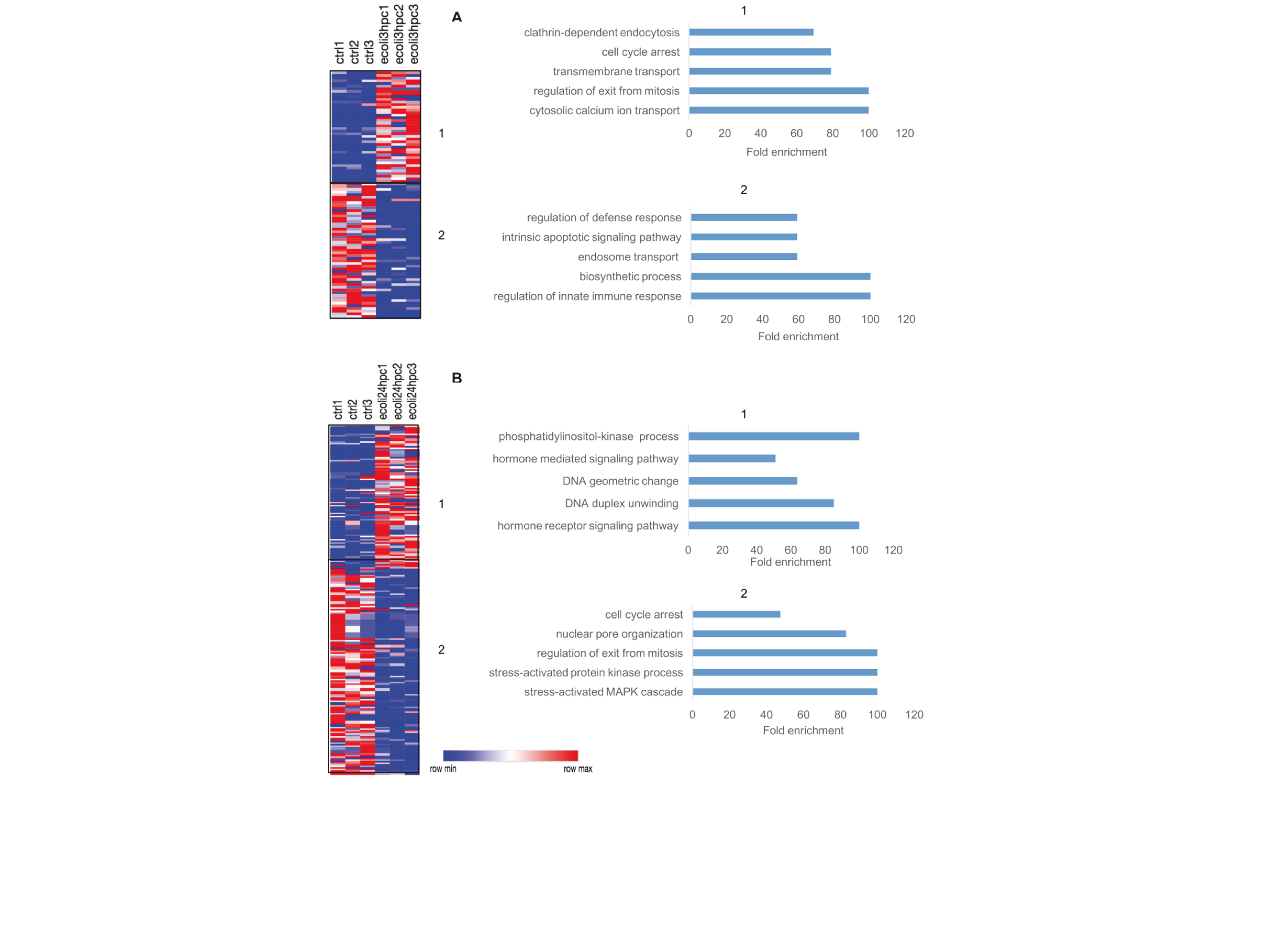

Supplement: Supplementary file 3 — Figure S3. k-means clustering of differentially methylated genes (CpG in TSS ± 2000) with k = 2 and scaled as Z-score across rows. Top five gene ontology (GO) biological processes derived from each k-means clusters ranked based on the fold enrichment. A) E coli 3 hpc vs control, B) E coli 24 hpc vs control. (TIF 2836 kb) [file 12864_2019_5976_MOESM3_ESM.tif]

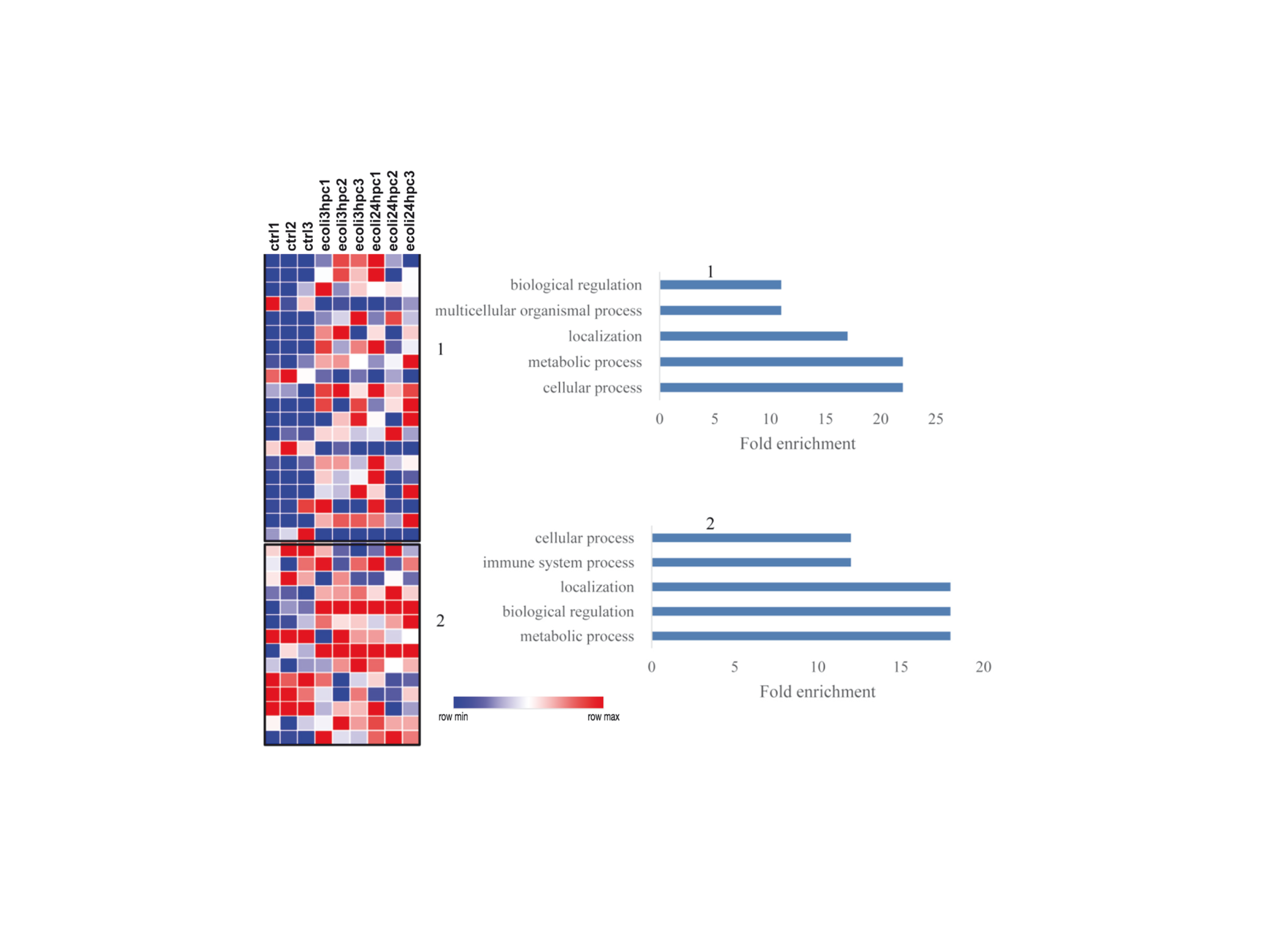

Supplement: Supplementary file 4 — Figure S4. k-means clustering of differentially methylated common CpG that were present in both E. coli 3 hpc vs control and E. coli 24 hpc vs control and top five enriched biological biological process. (TIF 1258 kb) [file 12864_2019_5976_MOESM4_ESM.tif]

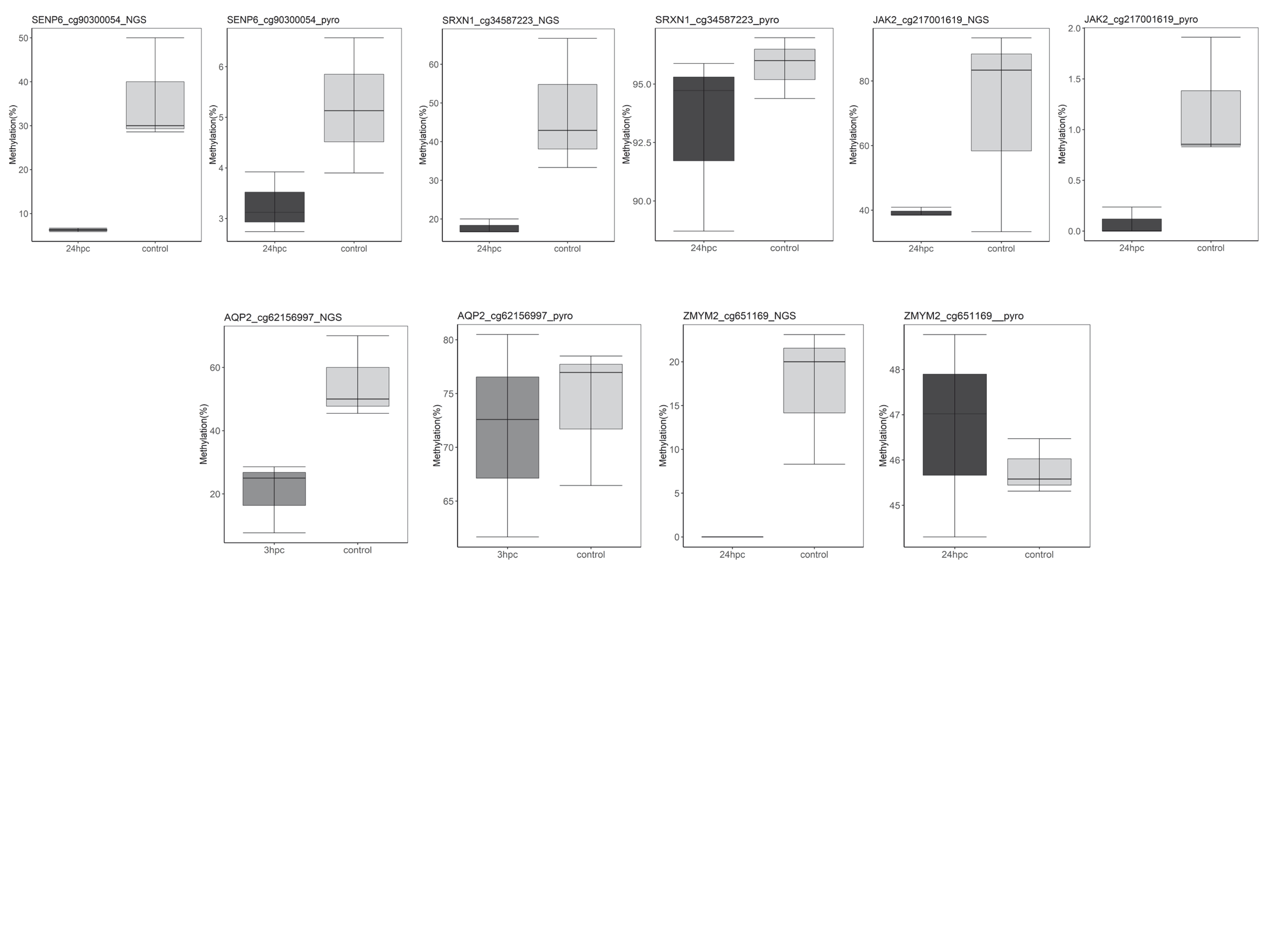

Supplement: Supplementary file 5 — Figure S5. Differentially methylated CpG sites identified between E. coli 3hpc or 24hpc compared to the unchallenged control group from NGS data compare to pyrosequencing including SENP6 (cg90300054), SDF4 (cg63545568), JAK2 (cg217001619), SRXN1 (cg34587223), ZMYM2 (c651169). The y-axis for both box plots represents methylation level. Genes associated with the CpG are given. Box plot represents the range of variation and median value. (TIF 2466 kb) [file 12864_2019_5976_MOESM5_ESM.tif]
