# Supplementary material for: DNA methylation analysis of porcine mammary epithelial cells reveals differentially methylated loci associated with immune response against Escherichia coli challenge
Source: BMC Genomics. 2019 Jul 31;20:623. doi: 10.1186/s12864-019-5976-7 (PMC6670134; doi:10.1186/s12864-019-5976-7)
Supplement: Supplementary file 9 — List of primers sequences used for quantitative gene expression and pyrosequencing. (DOCX 15 kb) [file 12864_2019_5976_MOESM9_ESM.docx]

Additional file 4. List of primers sequences used in quantitative expression analysis of genes and pyrosequencing for DNA CpG methylation

| **Primer Name** | **Sequence (5’ to 3’)** |
| --- | --- |
| SDF4_F | AGGGAGTTTGAGGAACTGATTG |
| SDF4_R | TCCAGGTAGTGGTTCTGGTTCT |
| SRXN1_F | TGGAATGAGAAAGGAGATGGTT |
| SRXN1_R | CCAGGTTTCCCTTTACTCACAG |
| CSF1_F | ACCATGCGCTTCAGAGACAA |
| CSF1_R | TGGAGGGGCGTCTCATAGAA |
| CXCL14_F | GAGGGAAAACGTCACACCGA |
| CXCL14_R | ACCCTCGGTAAAAGTGCTGG |
| ZMYM2_F | TTCCAGATGTCCTGTGAAAATG |
| ZMYM2_R | AGTGAAGCAGACGTGTACCAGA |
| DNMT1_F | TCAGGGACCACATCTGTAAG |
| DNMT1_R | GCTGCAGCCATTCTTCTTGT |
| DNMT3a_F | GGCTCTTCTTTGAGTTCTACCG |
| DNMT3a_R | GCGAGATGTCCCTCTTGTCA |
| DNMT3b_F | TGAAGAGTCCATCGCTGTTG |
| DNMT3b_R | CAATCACCAGGTCAAAGGG |
| BS_SENP6_F | GGTAGGAGGGTTATATAGAGGT |
| BS_SENP6_R-biotin | TTACTCRCCCTCAACCCCTCTT |
| BS_SENP6_seqF | TTTTTTTYGGAGAATG |
| BS_SRXN1_F | GTAGTGGTGGTAGGTGTTAAG |
| BS_SRXN1_R-biotin | AACCATCCCTACCAAACTTATC |
| BS_SRXN1_seqF | TGTTAAGGAGGTTGTTATT |
| BS_JAK2_F | GGAGAGTTTYGGAGGGTTTAGTT |
| BS_JAK2_R-biotin | CCCCCAAACTCACAAACAC |
| BS_JAK2_seqF | GGAYGTTYGGATTTTTGTTTT |
| BS_AQP2_F | TAGGGTAGAGATTYGAAAGAATTGAAGT |
| BS_AQP2_R-biotin | AATCCAATCRAAACCACAACTACCTAAC |
| BS_AQP2_seqF | TTTATTAGGATTTATGAGGATG |
| BS_ZMYM2_F | GGTTTTTATAAGGGGGATGTTG |
| BS_ZMYM2_R-biotin | ACCACCAAACCCTCTATCTATC |
| BS_ZMYM2_seqF | AGTATAGGTTTGGAGATTT |
